# Supplementary material for: Circularly polarised luminescence laser scanning confocal microscopy to study live cell chiral molecular interactions
Source: Nat Commun. 2022 Jan 27;13:553. doi: 10.1038/s41467-022-28220-z (PMC8795401; doi:10.1038/s41467-022-28220-z)
Supplement: Supplementary file 1 — Supplementary Information [file 41467_2022_28220_MOESM1_ESM.pdf]

Supporting Information

Circularly polarised luminescence laser scanning confocal microscopy to study live cell chiral molecular interactions

Patrycja Stachelek,[1,2] Lewis Mackenzie,[1,2] David Parker,[1] Robert Pal[1,*]

[1] Department of Chemistry, Durham University, South Road, Durham, United Kingdom, DH1 3LE.

[2] These authors contributed equally to the manuscript.

*Email of corresponding author: robert.pal@durham.ac.uk

Absorption and emission spectroscopy

Absorption spectra were recorded using ATI Unicam UV/Vis spectrometer (Model UV2) using Vision 3.33 software. All the steady-state emission spectra were recorded using ISA Jobin-Yvon Spex Fluorolog-3 luminescence spectrometer using DataMax v2.2 software. Samples were held within 1 cm path length quartz cuvettes.

CPL spectroscopy

PEM-CPL spectrometer:[1] CPL was measured with a home-built (modular) spectrometer. The excitation source was a broad band (200 – 1000 nm) laser- driven light source EQ 99 (Elliot Scientific). The excitation wavelength was selected by feeding the broadband light into an Acton SP-2155 monochromator (Princeton Instruments); the collimated light was focused into the sample cell (1 cm quartz cuvette). Sample PL emission was collected perpendicular to the excitation direction with a lens (f = 150 mm). The emission was fed through a photoelastic modulator (PEM) (Hinds Series II/FS42AA) and through a linear sheet polariser (Comar). The light was then focused into a second scanning monochromator (Acton SP-2155) and subsequently on to a photomultiplier tube (PMT) (Hamamatsu H10723 series). The detection of the CPL signal was achieved using the field modulation lock-in technique. The electronic signal from the PMT was fed into a lock-in amplifier (Hinds Instruments Signaloc Model 2100). The reference signal for the lock-in detection was provided by the PEM control unit. The monchromators, PEM control unit and lock-in amplifier were interfaced to a desktop PC and controlled by a custom-written Labview graphic user interface. The lock-in amplifier provided two signals, an AC signal corresponding to (I_L- I_R) and a DC signal corresponding to (I_L + I_R) after background subtraction. The emission dissymmetry factor was therefore readily obtained from the experimental data, as 2 AC/DC.

Spectral calibration of the scanning monochromator was performed using a Hg-Ar calibration lamp (Ocean Optics). A correction factor for the wavelength dependence of the detection system was

constructed using a calibrated lamp (Ocean Optics). The measured raw data was subsequently corrected using this correction factor. The validation of the CPL detection systems was achieved using light emitting diodes (LEDs) at various emission wavelengths. The LED was mounted in the sample holder and the light from the LED was fed through a broad band polarising filter and $\lambda/4$ plate (Ocean Optics) to generate circularly polarised light. Prior to all measurements, the $\lambda/4$ plate and a LED were used to set the phase of the lock-in amplifier correctly. The emission spectra were recorded with 0.5 nm step size and the slits of the detection monochromator were set to a slit width corresponding to a spectral resolution of 0.25 nm. CPL spectra (as well as total emission spectra) were obtained through an averaging procedure of several scans. The CPL spectra were smoothed using a shape-preserving Savitzky-Golay smoothing (polynomial order 5, window size 9 with reflection at the boundaries) to reduce the influence of noise and enhance visual appearance; all calculations were carried out using raw spectral data. Analysis of smoothed vs raw data was used to help to estimate the uncertainty in the stated gem factors, which was typically ±10%.

SS-CPL spectrometer:[2] Sample excitation was provided by a 2.35 W 365 nm LED with a 9 nm spectral half-width [CUN6AF4A; Roithner LaserTechnik] mounted in a custom-built heat sink. Power to the LED was supplied by a bench-top power supply [PL303QMD 30V/3A; Aim TTI] operating in constant current mode. A collimating lens, ground-glass diffuser [N-BK7, Thorlabs], and a 240 – 395 nm bandpass filter [FGUV5, Thorlabs] placed prior to the sample ensured that excitation light was diffuse, unpolarised, and constrained to $\lambda < 400$ nm. Samples were placed within an enclosed holder [CVH100, Thorlabs]. All samples were measured in quartz cuvettes with a 10 mm by 10 mm cross-section [111-10-40; Hellma Analytics]. Sample emission was collected 90° to the excitation beam. The emitted light passed through an achromatic QWP [AQWP05M-600; Thorlabs], which converted circularly polarised light into two orthogonal, linearly polarised, signals, corresponding to L-CPL and R-CPL. The light then was then split into two spatially separated detection channels by a non-polarising 50/50 beam splitter [BS013; Thorlabs]. Each detection channel was capable of independently measuring L-CPL and R-CPL in a sequential manner by precisely rotating a linear polariser [LPVISE100-A; Thorlabs] mounted in a motorized precision rotation mount [PRM1/MZ8; Thorlabs] controlled via an electronic controller [KDC101; Thorlabs]. A long pass filter ($\lambda > 450$ nm) [FEL0450, Thorlabs] prior to detection ensured no stray excitation light could reach the detector. Emission intensities of left CPL and right CPL channels were quantified by two matched high-sensitivity SS charge coupled device (CCD) spectrometer operating at 400 – 800 nm with ~0.2 nm sampling increments [Ocean Optics Maya2000Pro, H3 grating, 350 – 850 nm]. To maximise light throughput to the CCD detector, the spectrometer entrance aperture slit was removed.

From the intensity of each channel, the total emission $(I_{L-CPL} + I_{R-CPL})$, CPL $(I_{L-CPL} - I_{R-CPL})$ spectra and g_{em} (emission dissymmetry factor) $g_{em} = \frac{2(I_{L-CPL} - I_{R-CPL})}{(I_{L-CPL} + I_{R-CPL})}$ were calculated. Synchronised operation of both channels enabled rapid concurrent acquisition of full CPL spectra. SS-CPL operation was automated using custom-written LabVIEW programs [LabVIEW 2013, National Instruments].[3] Data was analysed post-hoc with custom-written Matlab programs [Matlab 2019a, MathWorks].

Time-resolved detection of whole-spectra was achieved by introducing a time delay between the pulsed LED excitation and the start of SS-CCD spectrometer acquisition. LED pulsation and spectrometer acquisition were synchronised to transistor–transistor logic (TTL) signals produced via a USB multifunction I/O device [USB-6210, National Instruments]. Acquisition at a rate of 43 Hz was found to minimize spurious noise (e.g. from mains frequency electronics at 50 Hz) whilst providing a suitable measurement window for time-resolved measurement of long-lived lanthanide emission (i.e. several milliseconds) with a 10-millisecond integration time.

MP-Spectroscopy

Two photon CPL spectroscopy has been achieved by coupling (beam routing using mirrors, Thor Labs BB1-E03) a tunable femtosecond pulsed laser (680 – 1300 nm, Coherent Discovery TPC, 100 fs, 80 MHz) to two pre-existing CPL spectrometers detailed above. Initial proof of concept two photon spectroscopy has been achieved by perpendicularly mounting an Ocean Optics HR2000Pro (2048-pixel linear CCD Sony ILX5 chip, 200 µm slit, H3 grating, 350 – 850 nm spectral region) spectrometer as a 'third arm' to the Discovery TPC laser. The laser beam was focused onto the centre of the 1cm path sample holder (Thor labs CVH100) by a dedicated ultrafast laser lens (Edmund Optics 11711, 50 mm focal length). The spectrometer has also been equipped with a perpendicularly mounted 365 nm LED (nichia, 1W) and been operated using a modified version of the above-mentioned custom time resolved detection and accumulation algorithm written in Labview2013 program.

In order to eliminate unwanted artefacts associated with stray light from MP excitation each spectrometer have been equipped with a rotating filter wheel (Thor Labs, CFW6) housing an LP420 (Comar Optics, for 365 nm UVLED excitation) and SP650 and SP700 (Edmund Optics, 8472 and 8474 for MP excitation) filters.

Cross section determination

The cross-sections (σ^2) of the standard materials discussed herein,[4,5] are calculated according to established procedures:[6]

$$\frac{\sigma_2^S \phi^S}{\sigma_2^S \phi^S} = \frac{C_R n_S F^S(\lambda)}{C_S n_R F^R(\lambda)}. \qquad \text{(eq.S1)}$$

Where S is sample, R is reference, ϕ is the total emission quantum yield of the compound (composite photoluminescence of both chromophore and Eu(III) ion for ϕ_S), C is the concentration, n the refractive index and $F^S(\lambda)$ and $F^R(\lambda)$ are the integrated PL spectrum for the sample and reference respectively. Additionally, we have demonstrated that the excitation process is definitely a two-photon event by recording an excitation power dependence; the resulting line has a slope of two on a logarithmic scale.[7] The cross sections were calculated with reference to Rhodamine B in ethanol. The MP-CPL spectrum was recorded using 6 µM solutions in 1 cm path length quartz cuvettes.

Spin coating procedure

Poly(vinyl-pyrrolidine) (PVP) ($M_w \sim 40,000$, Sigma-Aldrich) films were prepared as follows: the polymer is dissolved in methanol (MeOH) (55 mg/ml) and left stirring overnight at room temperature. The europium complex is dissolved in MeOH. 500 µl of the PVP solution was subsequently mixed with 100 µl of the lanthanide complex mixture. Three layers of the solution of the above mixture were deposited on a glass substrate at 1,500 RPM for 1 minute. The resulting film was annealed on a hot plate for 5 – 10 minutes at 35 ^{o}C after each layer was deposited to ensure there were no cracks on the surface of the film.

The resulting spin-coated films were of even thickness and quality that was confirmed by the observed homogeneous europium emission in the studied field of view (FOV) using laser scanning confocal microscopy (x40 0.7 NA objective, 0.3 AU pinhole, axial (z) resolution 300 nm). However, the edge of the glass slides were non-uniform and produced internal reflections that resulted in phase-inversion, i.e. destroying/interfering with the CPL signal. Therefore, only the bulk of these test-targets was considered suitable for the purposes of demonstrating CPL-LSCM, not the edges. Accordingly, they could not be used for side-by-side comparisons.

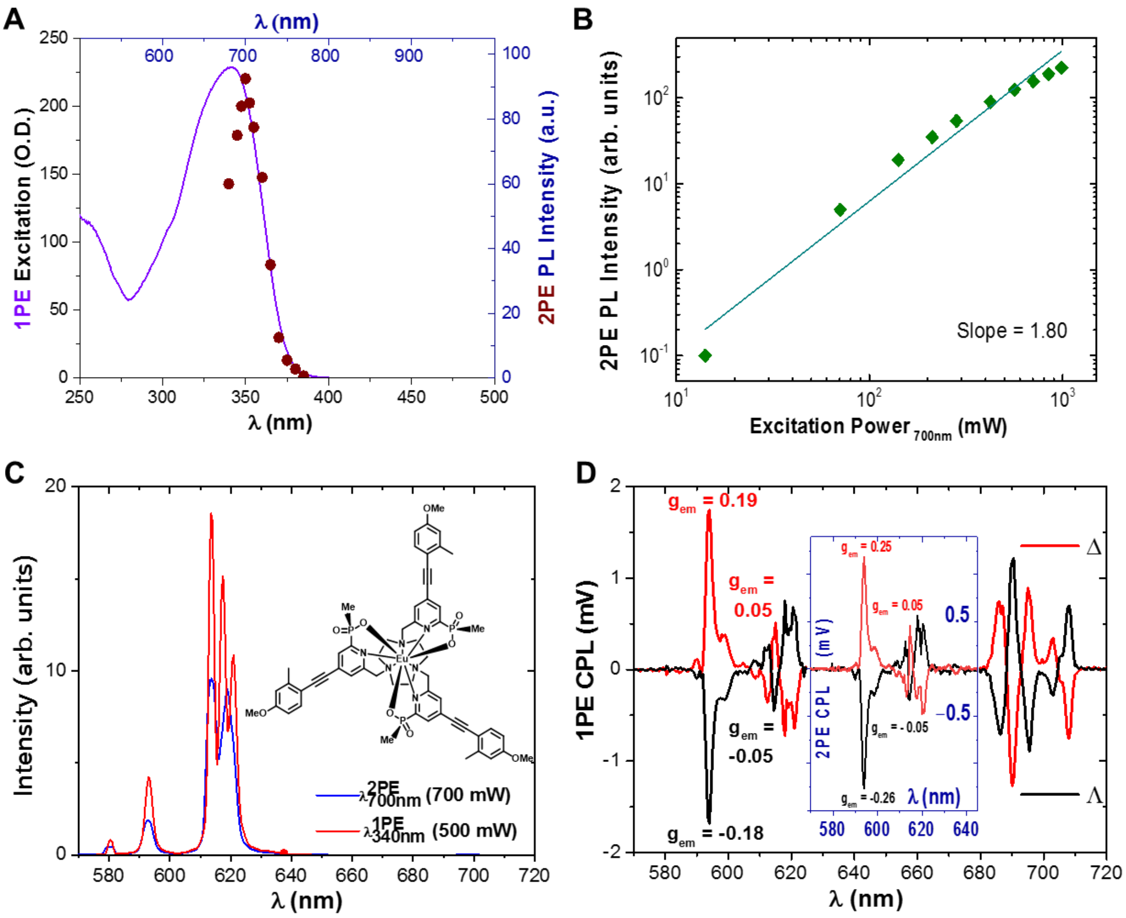

SI Fig. 1. Key photophysical parameters and spectra of Λ- and Δ Eu:L1 in MeOH. (A) One photon excitation (λ_{em} = 615nm) (solid purple line) and two photon (maroon dots) excitation spectra (λ_{em} = 615 nm) of Eu:L1. (B) excitation power dependency (green diamonds) of the 2PE photo luminescence (PL) intensity, slope 1.84 ± 0.1, σ^2= 51 ± 3 GM (10^{-50} cm^4s/photon). (C) One (λ_{ex} = 365 nm, solid red line) and two photon (λ_{ex} = 720 nm, solid blue line) induced emission spectrum of Λ- and Δ Eu:L1. Each enantiomer gave identical spectra. (D) One photon and (insert) two photon CPL spectra of Λ- and Δ Eu:L1 (solid black and red line respectively). All spectra in MeOH.

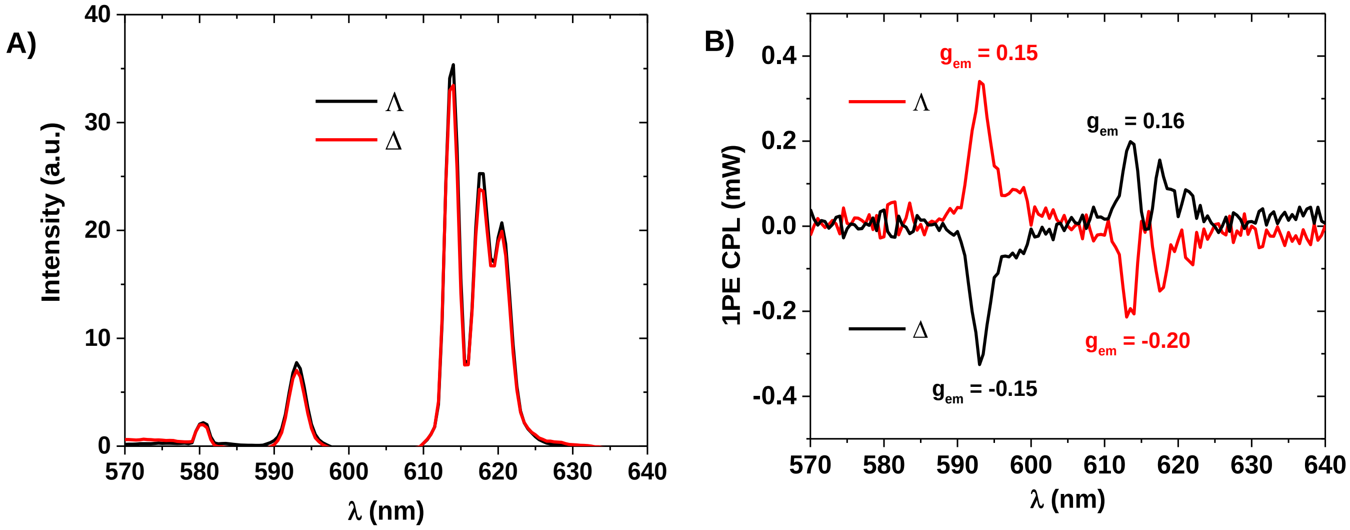

SI Fig. 2. Key spectra of Λ- and Δ Eu:L1 on glass substrate. (A) Total emission of Λ and Δ Eu:L1 drop-cast onto a 13 mm cover slip orientated at 45° to the excitation and collection paths ($\lambda_{ex.}$ = 340 nm, solid black and red line respectively. (B) One photon CPL spectrum of Eu:L1 encapsulated in a PVP film ($\lambda_{ex.}$= 355 nm to match the laser line used for CPL-LSCM, solid black and red line respectively). Data recorded with the conventional PEM-CPL spectrometer.

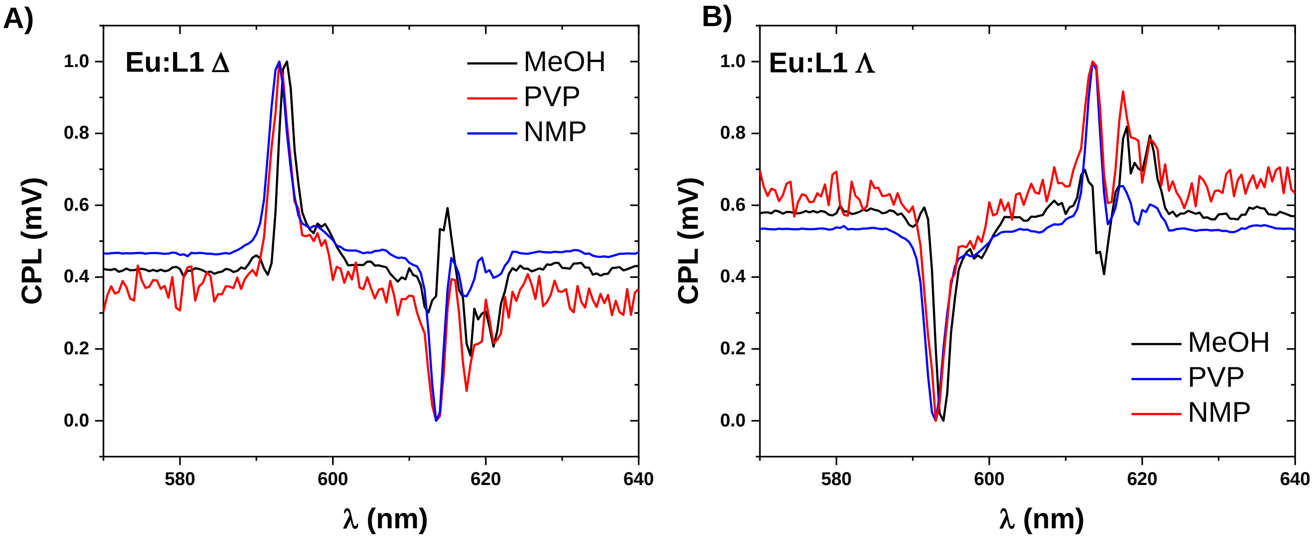

SI Fig. 3. Key spectra of Λ- and Δ Eu:L1 in relevant solvents and PVP. (A) One photon CPL spectra ($\lambda_{ex.}$= 355 nm) of Δ Eu:L1 in MeOH (solid black line) and N-Methyl-2-pyrrolidone (NMP) (blue) and Poly(vinyl-pyrrolidine) (PVP) matrix (solid red line). (B) One photon CPL spectra ($\lambda_{ex.}$= 355 nm) of Λ Eu:L1 in MeOH (black) and N-Methyl-2-pyrrolidone (NMP) (solid blue line) and Poly(vinyl-pyrrolidine) (PVP) matrix (red).

Live cell cultures studies

CPL - Laser scanning confocal microscopy experiments were conducted using NIH 3T3 embryonic mouse skin fibroblast cell line, sourced from ATCC (CRL-1658) and were established and maintained in a category 2 cell culture facility according to established standardized protocol for 12 months; they were periodically monitored for mycoplasma contamination.[8] Cells were maintained in exponential growth as monolayers in F-12/DMEM (Dulbecco's Modified Eagle Medium) 1:1 that was supplemented with 10% fetal bovine serum (FBS). Cells were grown in 75 cm^2 plastic culture flasks, with no prior surface treatment. Cultures were incubated at 37 °C, 10% average humidity and 5% (v/v) CO_2. Cells were harvested by treatment with 0.25% (v/v) trypsin solution for 5 min at 37 ºC. Cell suspensions were pelleted by centrifugation at 112 g for 3 min, and were re-suspended in fresh medium by repeated aspiration with a sterile plastic pipette. Microscopy cells were seeded in untreated iBibi 100 µL live cell channels and allowed to grow to 40% to 60% confluence, at 37 °C in 5% CO_2. At this stage, the medium was replaced and cells were treated with the studied nanomachines and co-stains as appropriate, with 0.1 % DMSO (as detailed above) present in the final imaging medium. For live cell imaging, DMEM/F12 media (10% FBS) lacking phenol red was used from this point onwards. Following incubation, where Method B was used, the channels were washed with live cell imaging media and imaged using a purpose build incubator housing the microscope maintaining 37 ºC, 5% CO_2 and 10% humidity.

Circular Polarisation Luminescence Laser Scanning Confocal Microscope (CPL-LSCM)

CPL-LCSM was enabled by adapting a commercial LSCM (SP5 II, Leica Microsystems) with excitation provided by a fibre coupled 80 mW variable power 355 nm Nd:YAG CW laser. The CPL analysis module was external to an output port and all elements were mounted in a 30 mm cage mount system for optimal alignment (assorted 30 mm components, Thorlabs). First, light from the sample focal plane excites the mirror controlled X1 emission port and passes through a high transmission bandpass filter (either 589 ± 5 nm or 594 ± 5 nm [Edmunds Optics, 65162 and 86737]) mounted in a switchable filter selector apparatus (CFS1/M, Thorlabs). These bandpass filters selected for emission from the ΔJ = 1 emission band of Europium. The circularly polarised emission is then converted to linearly polarised light by an achromatic quarter wave plate (AQWP05M-600, Thorlabs) and is separated into two detection arms by a simple 50/50 beam-splitter cube (BS013, Thorlabs). In each arm, the linearly polarised light is selectively analysed by linear polarisers (LPVISE100-A, Thorlabs) mounted within ultra-high precision computer-controlled

rotation mounts with ± 60 µrad unidirectional repeatability (K10CR1/M, Thorlabs), orientated to select for left or right CPL states via computer control software (Kinesis, Thorlabs). The intensity of emission in each path was quantified by fibre-coupled (200 micron) high performance matched tandem avalanche photodiodes (Leica ADPs, Becker & Hickl ID-120). The two detection arms were aligned to achieve matched sensitivity to enable rapid simultaneous acquisition of left and right CPL images. Calibration of the linear polarisers for enantioselective localisation was executed based upon the procedure reported in Mackenzie et. al.[2]

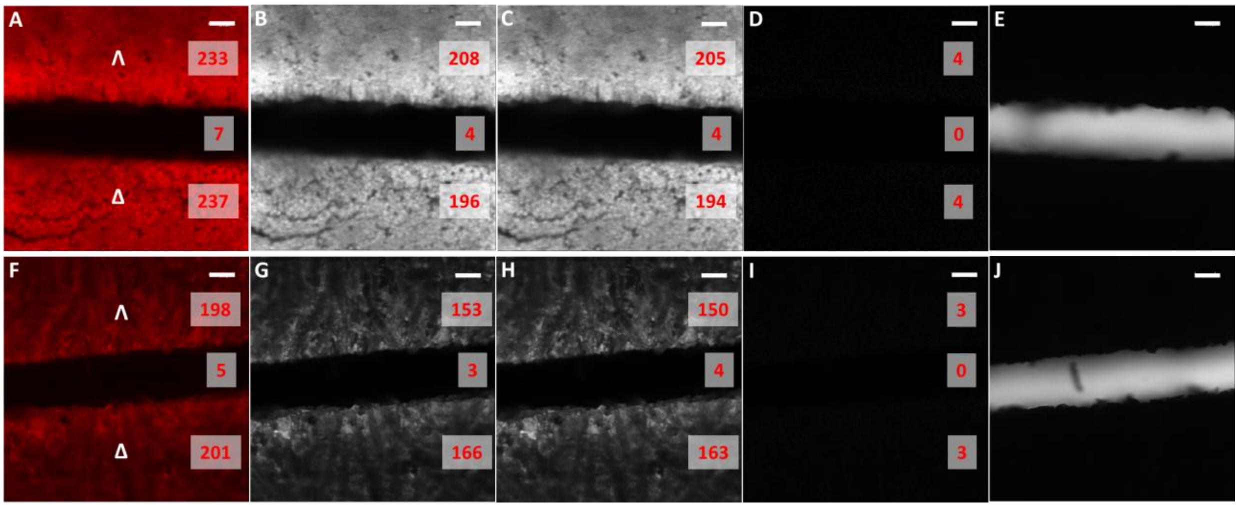

SI Fig. 4. Enantioselective differential chiral contrast (EDCC) CPL-LSCM of Λ- and Δ- Eu:L1 on paper substrate. Test Target 1 high axial resolution LSCM studies of enantiopure Eu:L1 blotted onto optical brightener free paper surface, Images A-E shows to the smooth side F-J shows to the rough side of the paper (A,F) Total Europium emission λex = 355 nm (Nd:YAG 3rdH, 20 mW), λem = 589-720 nm (B,G) Left Handed (C,H) Right Handed CPL channel λex = 355 nm, λem = 589-599 nm, (D,I) Differential chiral contrast image whereas Left handed CPL channel subtracted from Right handed CPL channel highlighting homogeneous zero difference in spatial distribution and brightness of the Eu-complex in the observed pinhole governed (3AU) axial section (4.5 µm) confirming unsuccesfull diferential chiroptical imaging. X40 0.7 NA air 100 image avg. x3 digital zoom, (E,J) transmission image (λex = 488 nm, 2 mW) scale bars represent 10 µm. Numbers in red indicate the mean 8 bit pixel intensity values for each image segment.

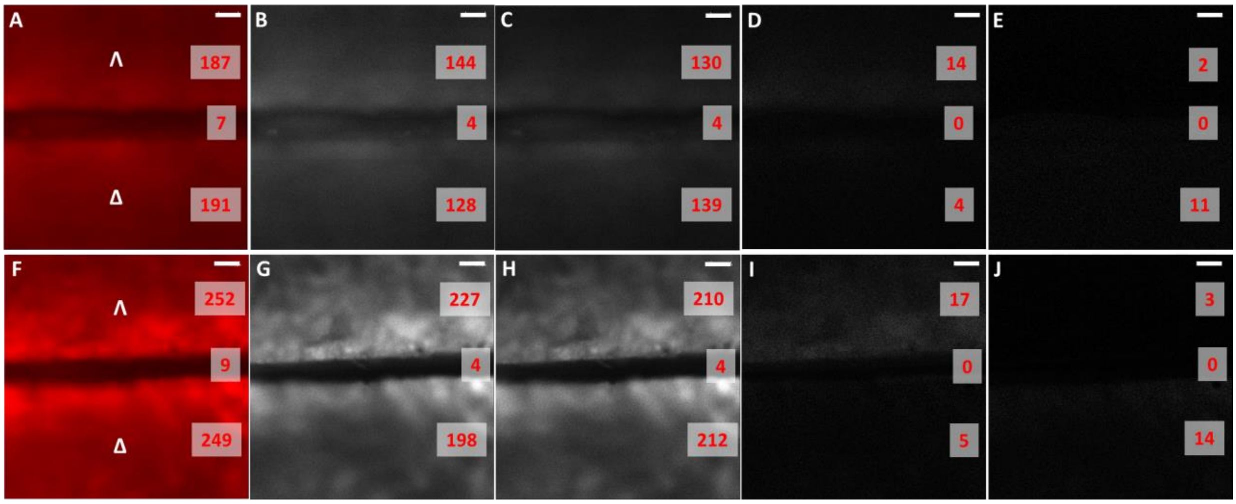

SI Fig. 5. Enantioselective differential chiral contrast (EDCC) CPL-LSCM of Λ- and Δ- Eu:L1 on paper substrate. Test Target 1 low axial resolution LSCM studies of enantiopure Eu:L1 blotted onto optical brightener free paper surface demonstrating succesfull diferential chiroptical imaging, Images A-E shows the smooth side F-J shows the rough side of the paper (A,F) Total Europium emission (λex = 355 nm, 20 mW), λem = 589-720 nm (B,G) Left Handed (C,H) Right Handed CPL channel λex = 355 nm, λem = 589-599 nm, (D,I) Differential image whereas Left handed CPL channel subtracted from Right handed CPL channel, (E,J) Differential image whereas Right handed CPL channel subtracted from Left handed CPL channel highlighting difference in brightness of the different enantiomers of Eu-complex in the observed pinhole governed (11 AU) axial section (17 µm). X10 0.4 NA air 100 image avg., scale bars represent 50 µm. Numbers in red indicate the mean 8 bit pixel intensity values for each image segment.

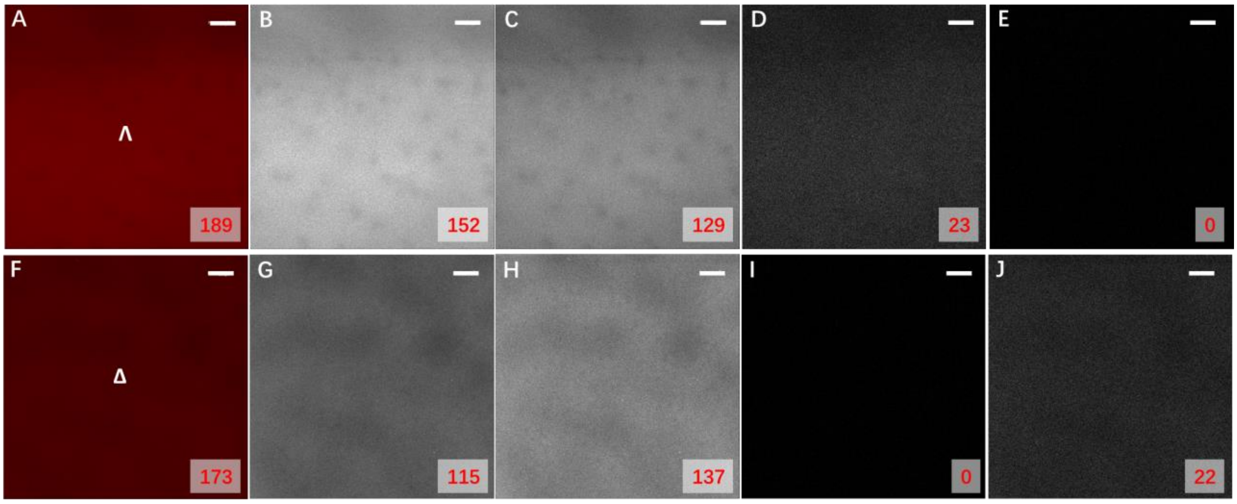

SI Fig. 6. Enantioselective differential chiral contrast (EDCC) CPL-LSCM of Λ- and Δ- Eu:L1 on paper substrate. Central region of Test Target 1 low axial resolution LSCM studies of enantiopure Eu:L1 blotted onto optical brightener free paper surface demonstrating succesfull diferential chiroptical imaging, Images A-E shows the blotted Λ enantiomer of Eu:L1 F-J shows blotted Δ enantiomer of Eu:L1 (A,F) Total Europium emission (λex = 355 nm, 20 mW), λem = 589-720 nm (B,G) Left Handed (C,H) Right Handed CPL channel λex = 355 nm, λem = 589-599 nm, (D,I) Differential image whereas Left handed CPL channel subtracted from Right handed CPL channel, (E,J) Differential image whereas Right handed CPL channel subtracted from Left handed CPL channel highlighting difference in brightness of the different enantiomers of Eu-complex in the observed pinhole governed (11 AU) axial section (49 µm). X10 0.4 NA

air 100 image avg., scale bars represent 50 µm. Numbers in red indicate the mean 8 bit pixel intensity values for each image segment.

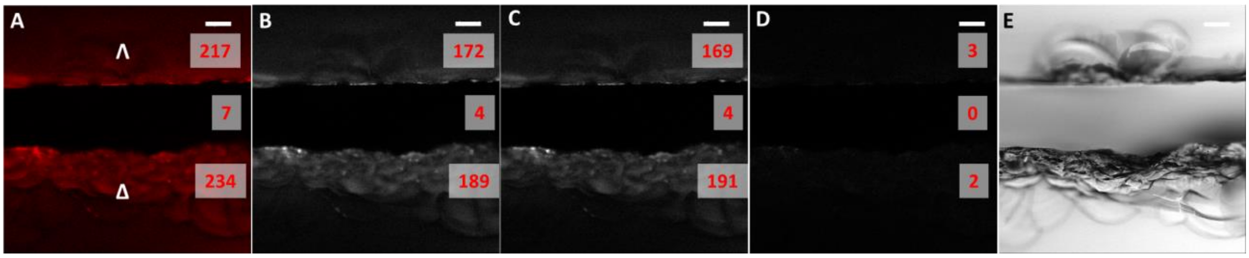

SI Fig. 7. Enantioselective differential chiral contrast (EDCC) CPL-LSCM of Λ- and Δ- Eu:L1 on glass substrate. Test Target 2 (A) Total Europium emission λex = 355 nm (20 mW), λem = 589-720 nm, simultaneously recorded (B) Left-Handed and (C) Right-Handed CPL channel (λex = 355 nm, λem = 589-599 nm) images of Test Target 1. (D) Differential image whereas Left handed CPL channel subtracted form Right handed CPL channel highlighting homogeneous spatial distribution and brightness of the Eu-complex confirming unsuccesfull diferential chiroptical imaging, (E) transmission image (λex = 488 nm, 2 mW) image highlighting the jagged edges that contribute to light helicity inversion due to internal reflections in the observed pinhole (5 AU) governed axial section (7.5 µm). X40 0.7 NA 100 image avg., x5 digital zoom, scale bars represent 10 µm. Numbers in red indicate mean 8 bit pixel intensity values for each image segment.

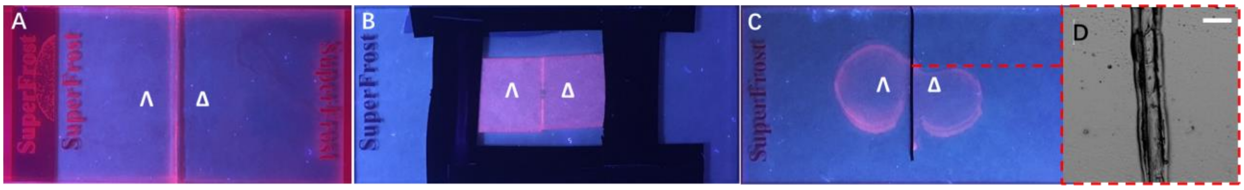

SI Fig. 8. Images of the test targets used throughout this study. Application and evolution of enantiopure Eu:L1 as test targets (A) Individually spin coated onto glass surface (B) individually blotted onto optical brightener free paper surface (C) simultaneously drop casted - separated by a non-fluroscent removable micro blade (20 µm) strip. All targets are illuminated with a 365 nm LED light source. (D) transmission image ((λex = 488 nm (2 mW) of the highlighted section after the micro blade has been removed and the sample has been capped by a 170 micron thick coverslip to facilitate high resolution (96 x 96 µm FOV, x63 1.4NA oil immersion) image acquisition and test target depth measurements to be executed, scale bars represent 30 µm.

References

1. Carr, R., Puckrin, R., McMahon, B.K., Pal, R., Parker, D. & Palsson, L-O., Induced circularly polarized luminescence arising from anion or protein binding to racemic emissive lanthanide complexes, *Methods Appl. Fluoresc*. **2**, 024007 (2014).
2. MacKenzie, L. E., Pålsson, L.-O., Parker, D., Beeby, A. & Pal, R. Rapid time-resolved Circular Polarization Luminescence (CPL) emission spectroscopy. *Nat. Commun.* **11**, 1676- (2020)
3. Pal, R & Beeby A. Simple and Versatile Modifications Allowing Time Gated Spectral Acquisition, Imaging and Lifetime Profiling on Conventional Wide-filed Microscopes. *Methods Appl. Fluoresc*. **2**, 037001 (2014).
4. Starck, M., MacKenzie, L. E., Batsanov, A. S., Parker, D. & Pal, R. Excitation modulation of Eu:BPEPC based complexes as low-energy reference standards for circularly polarised luminescence (CPL). *Chem. Commun.* **55**, 14115–14118 (2019).
5. Frawley, A. T., Pal, R. & Parker, D. Very bright, enantiopure europium(III) complexes allow time-gated chiral contrast imaging. *Chem. Commun.* **52**, 13349–13352 (2016).

6. Xu, C., & Webb, W.W., Measurement of two-photon excitation cross sections of molecular fluorophores with data from 690 to 1050 nm. *J. Opt. Soc. Am. B,* **13**(3), 481-491 (1996).
7. Pålsson, L.-O., Pal, R., Murray, B. S., Parker, D. & Beeby, A. Two-photon absorption and photoluminescence of europium based emissive probes for bioactive systems. *Dalt. Trans.* **6** 5726-5734 (2007).
8. Young, L., Sung, J., Stacey, G. & Masters, J. R. Detection of mycoplasma in cell cultures. *Nature Protocols*, **5**, 929-934 (2010).
