# Supplementary material for: Circularly polarised luminescence laser scanning confocal microscopy to study live cell chiral molecular interactions
Source: Nat Commun. 2022 Jan 27;13:553. doi: 10.1038/s41467-022-28220-z (PMC8795401; doi:10.1038/s41467-022-28220-z)
Supplement: Supplementary file 3 — Reporting Summary [file 41467_2022_28220_MOESM3_ESM.pdf]

nature portfolio

Corresponding author(s): Robert Pal

Last updated by author(s): Nov 11, 2021

Reporting Summary

Nature Portfolio wishes to improve the reproducibility of the work that we publish. This form provides structure for consistency and transparency in reporting. For further information on Nature Portfolio policies, see our Editorial Policies and the Editorial Policy Checklist.

Statistics

For all statistical analyses, confirm that the following items are present in the figure legend, table legend, main text, or Methods section.

n/a	Confirmed	
☐	☒	The exact sample size (n) for each experimental group/condition, given as a discrete number and unit of measurement
☐	☒	A statement on whether measurements were taken from distinct samples or whether the same sample was measured repeatedly
☒	☐	The statistical test(s) used AND whether they are one- or two-sided<br>*Only common tests should be described solely by name; describe more complex techniques in the Methods section.*
☒	☐	A description of all covariates tested
☒	☐	A description of any assumptions or corrections, such as tests of normality and adjustment for multiple comparisons
☒	☐	A full description of the statistical parameters including central tendency (e.g. means) or other basic estimates (e.g. regression coefficient) AND variation (e.g. standard deviation) or associated estimates of uncertainty (e.g. confidence intervals)
☒	☐	For null hypothesis testing, the test statistic (e.g. F, t, r) with confidence intervals, effect sizes, degrees of freedom and P value noted<br>*Give P values as exact values whenever suitable.*
☒	☐	For Bayesian analysis, information on the choice of priors and Markov chain Monte Carlo settings
☒	☐	For hierarchical and complex designs, identification of the appropriate level for tests and full reporting of outcomes
☐	☒	Estimates of effect sizes (e.g. Cohen's d, Pearson's r), indicating how they were calculated

Our web collection on statistics for biologists contains articles on many of the points above.

Software and code

Policy information about availability of computer code

Data collection	Leica LAS-X, NI Labview2013, JY DataMax v2.2, Unicam Vision 3.33, Matlab2019b
Data analysis	Image J v1.49h software have been used with its built in Calculator Plus add on to calculate differential images.

For manuscripts utilizing custom algorithms or software that are central to the research but not yet described in published literature, software must be made available to editors and reviewers. We strongly encourage code deposition in a community repository (e.g. GitHub). See the Nature Portfolio guidelines for submitting code & software for further information.

Data

Policy information about availability of data

All manuscripts must include a data availability statement. This statement should provide the following information, where applicable:

- Accession codes, unique identifiers, or web links for publicly available datasets
- A description of any restrictions on data availability
- For clinical datasets or third party data, please ensure that the statement adheres to our policy

The data generated and analysed during this study are available from the corresponding author upon reasonable request.

Field-specific reporting

Life sciences study design

All studies must disclose on these points even when the disclosure is negative.

Sample size	Each measurement have been repeated in triplicates and each sample have been recorded and analyzed independently in triplicates.
Data exclusions	No data have been exculded.
Replication	Each measurement have been repeated in triplicates and each sample have been recorded and analyzed independently in triplicates.
Randomization	The instrument and method development nature of our study and does not require or make randomization feasible.
Blinding	The instrument and method development nature of our study does not require or make blinding feasible.

Reporting for specific materials, systems and methods

We require information from authors about some types of materials, experimental systems and methods used in many studies. Here, indicate whether each material, system or method listed is relevant to your study. If you are not sure if a list item applies to your research, read the appropriate section before selecting a response.

Materials & experimental systems

n/a	Involved in the study
☒	☐ Antibodies
☐	☒ Eukaryotic cell lines
☒	☐ Palaeontology and archaeology
☒	☐ Animals and other organisms
☒	☐ Human research participants
☒	☐ Clinical data
☒	☐ Dual use research of concern

Methods

n/a	Involved in the study
☒	☐ ChIP-seq
☒	☐ Flow cytometry
☒	☐ MRI-based neuroimaging

Eukaryotic cell lines

Policy information about cell lines

Cell line source(s)	NIH 3T3 cell line was sourced form ATCC - CRL-1658
Authentication	ATCC's STR protocol and report
Mycoplasma contamination	Periodic test have been conducted on the NIH 3T3 cell linesas a routine laboratory protocol before each individual experiment. The NIH 3T3 cell lines used tested negative throughout the study.
Commonly misidentified lines (See ICLAC register)	No commonly misidentified cell lines have been used.
